# The New York Homœopathic Union

**Published:** 1895-03

**Authors:** Emma D. Wilcox

**Affiliations:** Secretary


### THE NEW YORK HOMŒOPATHIC UNION.

The regular monthly meeting of the New York Homœopathic Union was held at 53 W. Forty-fifth Street, New York, December 15th, 1894, the President, Edmund Carleton, M. D., in the chair. There were present Drs. Baylies, Alice B. Campbell, John B. Campbell, Fincke, Finch, O'Brien, Powell, Rushmore, Thomson, and Wilcox; also Medical Student George W. Willcox.

The following report is a very much condensed statement from the proceedings of a very long and most interesting meeting:

The reading of Hahnemann's *Chronic Diseases* was resumed, beginning on page 22.

Dr. Finch—Does Hahnemann mean that all ailments that are continuous come from psora? If so, then does it not follow that even where people have never had the itch their complaints can be traced back of them to the itch?

Dr. Thomson—Hahnemann says that itch may be internal or communicated.

Dr. Finch—Does not that expression seem rather to be a convenient one?

The President—Dr. Finch's questions bring us right to the centre of a very important matter; and right here let me quote the comments of Raue in the first edition of his work on special pathology:

"The old school considered it a great triumph when, in the year 1834, by M. Renucci, a young Corsican in Paris, who had learned in his native island the art of extracting the little animal, the question about the nature of itch seemed to be settled. Hahnemann's psora theory had thus been exploded by a needle in a Corsican's hand, and with it all Homœopathy! They simply forgot, in their hearts' delight, that before that time many other cutaneous eruptions were considered as itch; amongst them, as Hebra himself supposes, pruritus, with its undoubted metastases to inner organs. If we now take a glance over Hahnemann's masterly picture of what he calls psora, we shall at once perceive that, under psora, he did not understand acarus-itch solely, but gave a *tout ensemble* of chronic cutaneous affections in general. The child had to have a name, and psora was as good a name as eczema, impetigo, prurigo, or any one else. Thus the needle, although it found the acarus, missed altogether Hahnemann's psora.

"It is just as true to-day that a suppression of cutaneous eruptions of various kinds will be followed by disastrous consequences upon the general system, as it was true when Hahnemann and others observed it. Instead, then, of desiring to have Hahnemann's psora theory wiped out of the pages of Homœopathy as a disgraceful spot, we ought to be proud of our old master's keen observation. But then we must understand him rightly! I admit that, in recent cases of acarus-itch, the killing of the animal is the shortest procedure to cure without detrimental effects upon the organism. This end may be attained not only by the external application of *Sulphur and mercurial salves*, but also by *Peruvian Balsam*, or the *twigs of the balsam poplar tree* (*populis balsamifera*, L.; *tacamehac*, Ind.), which secretes a kind of resinous substance on the pedicles of its leaves and around its twigs. But, as it is an undoubted fact that *itch never heals spontaneously,* and as we have likewise undoubted facts that itch has been cured solely by the internal application of homœopathic remedies, it seems that those who contend that even acarus-itch in the course of time is not altogether a mere local, cutaneous trouble, are, after all, deserving some credit. All parasites, no matter whether animal or vegetable, can grow only upon a suitable soil. If this soil be made insupportable to them, they die or leave, and this is as good as killing, regarding the riddance of the intruders; but it is infinitely better for the patient, as by this means the organism is not injured, but brought into a healthy state."

Dr. Thomson—A *healthy* child will not catch the itch. No disease can be taken unless the system is susceptible to it; and the healthy system is not susceptible to itch.

Dr. Fincke—Three students inserted each a female acarus under the epidermis. The passage formed by the insect could be seen as it branched out without itching or vesicles. Suddenly, in one night, the eruption appeared under violent itching, upon the whole body. "There is the proof that the acarus is the itch," they said. But how could the single acarus even with her descendants travel over the whole body in one night without a sign of vesication and itching before?

In a case of itch-eruption all over the body no acarus could be found, but the greater part of the family was infected.

Four homœopathic physicians produced the itch by transferring the serous contents of the itch-vesicles.

Dr. Kunkel, to whom we owe these facts, concludes from them that there is a constitutional sickness which deposits its contagium in form of itch upon the skin. From various writings of Hahnemann it appears that he knew of the acarus, though he never mentioned it by name, but spoke of a living miasm [*I. H. A.*, 1893, p. 85].

Dr. Baylies—I do not use the same remedy in all cases, but look to the individual symptoms showing the susceptibility, and these being removed the soil is then purified and incapable of fostering the acarus. Is it not odd that Hahnemann is so strong against any but internal treatment in psora, and yet recommends Camphor externally as well as internally in cholera?

Dr. Thomson—But remember the reason. There among the peasantry the air was so surcharged with filth and stench that the Camphor was necessary to penetrate the air and clear it of other odors, thus rendering the action of the internal remedy efficacious.

Dr. Rushmore—Where does this infection come from?

Dr. Thomson—Correspondential susceptibility of the ailment is dormant in the patient, and is brought out by some excitement or strain, whether atmospheric, moral, or mental.

Dr. Baylies—You do not mean that we are Pandora's box filled with the seeds of all ailments.

Dr. Thompson—No; but each is susceptible in his own way to his own kind.

Dr. Finch—Go to science, and Tyndall tells us that no life can come without antecedent life, though the antecedent may be in the germ.

Dr. Thomson—Yes; *Ex nihilo nihil fit.* Nothing can come without a cause; but that cause need not of necessity be material.

Meeting adjourned.

EMMA D. WILCOX,
*Secretary.*

